# Balance and Coordination Improvements in Children and Adolescents with Autism Spectrum Disorder (ASD), Resulting from a Hydrotherapy Intervention

**DOI:** 10.3390/children13010094

**Published:** 2026-01-08

**Authors:** Meir Lotan, Marc Weiss

**Affiliations:** 1 Physical Therapy Department, Faculty of Health Sciences, Ariel University, Ariel 40700, Israel; marc.weiss@msmail.ariel.ac.il; 2 Buot Hydrotherapy Center, Ashdod 7724004, Israel

**Keywords:** autism spectrum disorder (ASD), autism, Hydrotherapy, balance, coordination, movement assessment battery for children -second edition (M-ABC-II), goal attainment scale (GAS)

## Abstract

**Highlights:**

**What are the main findings?**

- Hydrotherapy intervention improves motor challenges in children and adolescents with ASD.
- Hydrotherapy intervention improves communication, independence, as well as other core issues in children and adolescents with ASD.

**What are the implications of the main findings?**

- Hydrotherapy should be considered as a common therapeutic possibility for children and adolescents with ASD.

**Abstract:**

Background/objective: Despite the fact that almost 87% of children with Autism Spectrum Disorder (ASD) have physical coordination issues, motor skills are not the primary concern when ASD is diagnosed. An aquatic environment can provide multisensory stimuli that might assist these children; however, studies related to hydrotherapy with children with ASD have not yet examined whether this environment has an effect on balance and coordination. Methods: A control vs. research group examined the effect of a weekly, three-month hydrotherapy program on the balance and coordination abilities of male children and adolescents diagnosed with high-functioning ASD. Children (N = 22) between the ages of 6 and 17 years (mean: 8.4 ± 2.4), participated in this study. Each participant’s coordination and balance abilities were evaluated using the Movement Assessment Battery for Children-Second Edition (M-ABC-II). The initial evaluation (test one) was repeated (test two) after two months to establish improvement prior to intervention. The final evaluation (test three) was conducted at intervention termination. Individual functional goals were set for each patient using the Goal Attainment Scale (GAS). Results: No improvement was noted within the pre-intervention period (between tests one and two), yet there was a statistically significant improvement in the M-ABC-II Total Test score (*p* = 0.0133), in Manual Dexterity (*p* = 0.0181), and balance (*p* = 0.0053) post-intervention, between tests two and three. The mean GAS score for this study was 52.1, suggesting the achievement of prespecified functional goals. Conclusions: This study demonstrated a positive impact of a 12-week hydrotherapy program on balance and coordination and manual dexterity among children with ASD. A positive impact was also noted in patients’ individual functional abilities.

## 1. Introduction

Autism Spectrum Disorder (ASD) is a complex developmental condition that poses motor challenges, as well as the better-known social/emotional, speech and nonverbal communication challenges [1]. The incidence of ASD is constantly rising, with incidence reported recently by the Centers for Disease Control (CDC) at 1:31 children [2]. Since the main focus of therapeutic interventions in ASD is on behavioral, emotional, and communicational issues, scarce research has been conducted to date on the influence of aquatic treatments on challenges in the motor realm. Furthermore, studies related to hydrotherapy that have been performed in children diagnosed with ASD have not examined the effect of hydrotherapy on balance and/or coordination [3,4,5,6,7,8,9].

Most children with ASD (86.9%) have physical coordination issues [10]. Despite such high prevalence, motor skills are not the primary concern when ASD is diagnosed, and only 31.6% of children with ASD receive physical therapy services [11]. Children and adults with ASD (aged from 7 to 32 years) have shown poor upper-limb coordination during visuomotor and manual dexterity tasks, and poor lower-limb coordination during tasks requiring balance, agility, and speed [12]. Difficulties in motor skills include poor upper-limb and lower-limb coordination, including bilateral coordination and visuomotor coordination [12]. Moreover, the literature suggests that there are correlations between the severity of motor difficulties presented by children with ASD, and the severity of ASD; therefore, children with poorer motor skills have greater deficits in social communicative skills [13]. The impact of motor difficulties in childhood may also contribute to reduced participation with peers during play and sports, and, as a result, may further hamper social interaction and social development [11,14]. Bhat et al. propose that early motor delays within the first two years of life may contribute to the social impairments of children with ASD [12]. These primary and secondary difficulties highlight the need for early, intensive, appropriate therapeutic intervention for this group of children. Therefore, motor impairments should be addressed early, with the use of timely assessments and appropriate and effective interventions [12]. One of the suggested therapeutic approaches for this group of patients is hydrotherapy.

Hydrotherapy is the use of water in any of its forms (water, ice, steam) for health promotion or treatment of various diseases. It is one of the naturopathic treatment modalities used widely in ancient cultures, including India, Egypt, China, as well as others [3]. Hydrotherapy incorporates exercises (active and passive) performed in water and takes advantage of the effects of water on the body (e.g., temperature, density, buoyancy, hydrostatic pressure).

According to the American Physical Therapy Association [3], hydrotherapy provides stability to the patient’s body, enhanced ease of movement, enhanced proprioceptive awareness, and enhanced sensory feedback. Hydrotherapy’s positive effects on the human body in populations other than ASD were found to include relaxation [15,16], improved ability to participate in training exercises, high-intensity exercise and greater comfort [17,18], motor control, and functional mobility and swimming skills [19,20].

An aquatic environment provides stability and uniform sensorial support, allowing ease of movement and improved body awareness [5,11], which in turn allows an individual to practice important movement skills with fewer body constraints. Therefore, children with movement difficulties may experience more success in attaining movement skills in an aquatic environment [11].

Studies on children with ASD show increasing evidence that hydrotherapy may be effective in improving core elements of ASD, such as social interactions, communication skills, sensory processing, behavior [5,7], as well as aquatic skills [5,8,9], physical fitness [11], object control, and locomotor skills [6]. Yet, only few of those studies focused on motor elements such as balance [21], yet most of the recent meta-analysis performed in relation to motor achievements related to hydrotherapy intervention with children with ASD report methodological issues and heterogeneity between different studies, enabling but little scientific rigor regarding out-of-water motor achievements [22,23].

The most reliable assessments [24] and commonly used assessment tools for motor assessment in children with neurotypical development [25], which also include the most psychometrically suitable motor assessment tools for children with ASD [26] are: Movement Assessment Battery for Children—(MABC-2) [24,26], Test of Gross Motor Development, Second Edition—(TGMD-2) [24,27], Peabody Developmental Motor Scales, Second Edition—(PDMS-2) [28] and Bruininks–Oseretsky Test of Motor Proficiency, Second edition—(BOT-2) [29]. All of these scales have been found with good psychometric values for neurotypical children as well as for children with disabilities [26,29] and have been previously used in scientific papers assessing motor abilities of children with ASD.

PDMS-2 (aged 0–7 Y) and TGMD-2 (3–10 Y) could not be used as they do not cover the full age range of possible participants within the current study (6–16 Y). The BOT-2 covers the age range of participants within the current study but requires 45-60 min to be fully administered [29], while MABC-2 takes only minutes to be administered [29]. Therefore, the MABC-2 was selected to be the preferred motor scale in the current study as it covers the full age range of the participants (6–16 Y) and is the most frequently used by examiners to test the gross motor performance in children [24].

While current research suggests that hydrotherapy can improve gross motor skills among children diagnosed with ASD [3,4,5,7,8,9], these studies focused mainly on aquatic skills while mostly ignoring issues related to transfer of aquatic intervention into land skills, such as balance and coordination. Therefore, we found this topic appropriate for the current research project.

## 2. Methods

Ethical approval statements: The study was conducted in accordance with the Declaration of Helsinki. The research was conducted after receiving ethical permission from the Ariel University IRB (AU-HEA-ML-20231011) on 11 October 2023and authorization from the Buot hydrotherapy center’s management; all parents and some of the participants (Diagnosed with high-functioning ASD) signed an informed consent form.

Research goal: Examine the effect of a once weekly three-month hydrotherapy program on the balance and coordination abilities of children and adolescents with ASD.

Research hypothesis: A three-month hydrotherapy program will significantly improve the motor skills of balance and coordination among children and adolescents diagnosed with ASD.

Methods: This study comprised a control group (children not receiving hydrotherapy intervention) and the same children served as their own comparison group (study group), which was identical (same children) to the study group, thereby eliminating clinical heterogeneity typical of those with ASD as they both were the same individuals, once measured without the therapeutic intervention (control), and once after receiving the therapeutic intervention (study group). The pre–post method, which uses the participants as their own control group (with and without treatment), is consistent with previous studies conducted on this population and accounts for considerable inter-variability between ASD patients [4,5,7,8,13].

Participants: The study included 22 male participants between the ages of 6 and 17 years (mean, 8.4 ± 2.4), who were diagnosed with high-functioning ASD, according to the DSM-5 (level 1) or ADOS-II (mild ASD), and ability to understand simple instructions and comply with these instructions during a hydrotherapy session. (see Table 1 for demographic details and scores.)

### Inclusion and Exclusion Criteria

Inclusion Criteria: Male children (aged 6–17 years) diagnosed with ASD according to ADOS or ADI-R, demonstrating full cooperation and presenting an average M-ABC-II score below the 15th percentile (see extended explanation below), and able to understand and follow instructions.

Exclusion Criteria: Children who suffer from an additional severe chronic medical disorder in addition to ASD (e.g., neurological, psychiatric, metabolic, genetic, or orthopedic disorder), children with hearing impairments as a result of acquired trauma, children who take anti-anxiety medications, children in the middle of physical therapy or any type of intervention meant to improve balance and coordination (including hippotherapy and hydrotherapy), children who have difficulty cooperating and could not fully cooperate with the researcher’s instructions, and children presenting fear of water (based on the Humphries Assessment of Aquatic Readiness [HAAR] measure).

## 3. Procedure

**Figure 1:**
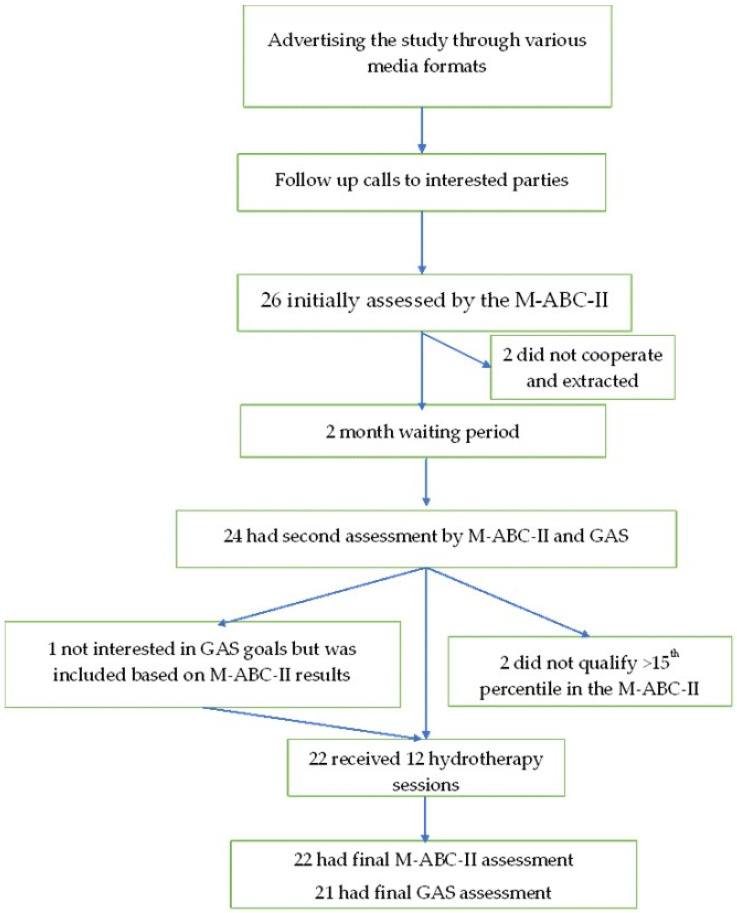
Flowchart of recruitment and intervention.

Recruitment and Baseline Assessments: (presented visually in Figure 1): Only children officially diagnosed with ASD were recruited to participate in the study. This was performed by advertising on social media platforms, through the social services departments in the municipalities of the cities Ashdod and Ashkelon, with the help of colleagues, and directly through Buot Hydrotherapy Center.

After initial contact was made, interested families signed informed consent forms, and an initial evaluation using the M-ABC-II was chosen to determine a baseline score. All evaluations were conducted by the researcher (the first author, M.W.) in the offices at Buot Center. After the initial evaluation, a date was immediately set for the second evaluation two months later. The time frame of a two-month waiting period, where no intervention was conducted, was chosen based on the clinical experience of the authors conducting the study in order to establish a comparison between the control (no treatment) and the study (with treatment). On completion of the second evaluation, the scores were averaged to establish a steady baseline score, percentile rank, and level of function. Participants were included in the research study if their average score was below the 15th percentile on M-ABC-II. Personal goals were established, while consulting the participants and their families, using the Goal Attainment Scale (GAS) after the baseline assessments and before the commencement of treatment sessions in the water. All evaluations were recorded for review by an independent and objective professional (an occupational therapist who has many years of experience implementing the M-ABC-II).

Each participant received a three-month hydrotherapy weekly intervention, as was performed in previous intervention protocols with children with ASD (4,8,9), with each therapeutic session lasting 30 min and divided into two equal parts, focusing on the two specific aspects of the study goals (balance and motor coordination). Minor adaptations were made to accommodate the individual needs and interests of each participant. In each therapeutic section, each exercise moved from a lower to a higher difficulty level after the participant had achieved control over the previous level (see Supplemental Materials—Therapeutic protocol of the hydrotherapy program).

The treatment was conducted by the Primary Investigator (PI. MW), an experienced pediatric physical therapist working as a licensed hydro-therapist. Each participant was exposed to an intervention program designed and constructed by the researchers, based on physical therapy principles used in the treatment of children in a clinical setting, combined with the principles of treatment in hydrotherapy to address the goals of the program. The plan and structure of the hydrotherapy intervention were based on protocols previously used in hydrotherapy for children with ASD, as well as the experience of the PI, who specialized in the field of early intervention and pediatric neuromuscular disorders for 27 years and who has been working as a hydro-therapist for the past 10 years, with various populations, including children with ASD. Hydrotherapy sessions were provided once weekly for 30 min, including exercises that focused on balance and coordination that progressed to higher levels of difficulty, depending on how each participant responded. At the end of the 12 session series, a final evaluation was conducted using the M-ABC-II. The individual goals achieved were evaluated through the use of the GAS. The final evaluations were also recorded for review by an independent and objective professional.

## 4. Outcome Measures

### 4.1. Movement Assessment Battery for Children

Movement Assessment Battery for Children—Second Edition (M-ABC-II): The M-ABC-II is a valid and reliable assessment of fine and gross motor skills [30,31,32] that has been used in children diagnosed with ASD [14,22]. The M-ABC-II includes eight tasks within three domains—manual dexterity, ball skills (aiming and catching), and static and dynamic balance. The strengths of the M-ABC-II include its ability to assess children using a different combination of fine and gross motor skill items appropriate for three different age groups within the age range from 3 to 16 years (age bands [AB]): AB1, three–six years; AB2, 7–10 years; and AB3, 11–16 years [31]. Within studies that use the M-ABC-II, children vary in age, but they are mainly within AB1 and AB2 [32,33,34,35,36]. The duration of a full assessment is in the range of 20–40 min, depending on the age of the child, the level of the child’s functioning, the cooperation of the child, and the skill of the examiner. One study showed high intra- and inter-rater reliability in the M-ABC-II performed in children in AB 2 [36]. In a study conducted on Thai children, inter-rater reliability was moderate to good (intra-class correlation coefficient of inter-rater reliability ranged from 0.71 to 1.00) [35]. Each item within the M-ABC-II is assigned a raw score; within each domain, the raw scores of the items are summed to reach a raw domain score, which is converted into a standard score and corresponding percentile. The scores for each of the three domains are summed into a standardized total score and percentiles. The percentiles are further classified: ≤5th percentile is the red zone, indicating significant movement difficulty; a score between the 5th and 15th percentile is classified as the amber zone, indicating being at risk of movement difficulty; and a score > 15th percentile is classified as the green zone, indicating no movement difficulty detected. The M-ABC-II presents high psychometric values, with test reliability found to be moderate to high [31]. One study demonstrated low sensitivity (41%) and acceptable specificity (88%) [32], while another study showed that the M-ABC-II at age 4 years had high sensitivity (79%) and specificity (93%) for predicting motor impairment at age 8 years [25]. The M-ABC-II has been tested in numerous populations with various functional disabilities, including ASD [22]. In this study, which included 30 children with ASD (age 3–16 years; 25 males, 5 females), the authors concluded that children with ASD would be delayed in fine and gross motor skill development, compared to their age-matched typically developing peers [31].

In the current study, the PI administered all of the tasks according to the M-ABC-II manual, including detailed verbal descriptions and demonstrations prior to the participants’ motor skill performances. To reduce the possibility of bias from the PI during M-ABC-II administration, each exam was recorded and reevaluated by an experienced professional (an OT working with children with ASD for over 15 years, trained in the use of the M-ABC) blinded to the time and place of the test within the research timeframe. The mean score of the PI and professional was used for statistical analysis. The primary focus of the treatment sessions was to improve balance and coordination; therefore, the score in the category of Balance was the main focal point, and therefore, the treatment program was designed for that purpose as well. Secondary analysis included the other domains of the M-ABC-II as well as the Total Test score.

### 4.2. Goal Attainment Scaling

Goal attainment scaling (GAS) is a method of scoring the extent to which one achieves individual goals throughout the intervention [37]. Each patient has their own outcome measure that is scored in a standardized way to allow for statistical analysis. Traditional standardized measures include a standard set of tasks/items, each rated on a standard level. In GAS, goals are individually established to suit the patient (and are therefore highly appropriate for children with ASD presenting vast clinical heterogeneity), and therefore, the levels are individually set around their current (pre-intervention) and expected (post-intervention) levels of performance. An important feature of GAS is the “a priori” establishment of criteria for a “successful” outcome in each individual, which is agreed upon by the patient and family before the intervention starts. Each goal is rated on a 5-point scale, with the degree of attainment captured for each goal area:

If the patient achieves the expected level, this is scored at 0. If they achieve a worse-than-expected outcome, this is scored at −1 (somewhat worse) or −2 (much worse/no change from baseline). If they achieve a better-than-expected outcome, this is scored as +1 (somewhat better) or +2 (much better).

Goals may be weighted (weight = importance × difficulty) to account for the relative importance of the goal to the participating individual and/or the anticipated difficulty of achieving the goal. A final score of approximately 50 represents a high-quality GAS outcome and indicates that functional goals were achieved. A final score below 50 implies that the set goals may have been too difficult to achieve, while a score over 50 implies that the goals were too easy to achieve [37].

The set goals followed the SMART principles (i.e., Specific, Measurable, Attainable, Realistic, and Timely).

### 4.3. Social Responsiveness Scale II—(SRS-2)

The SRS-second edition [38] is a 65-item questionnaire, measuring the severity of autism spectrum symptoms as they occur in natural social settings. The scale is completed by parents, and it is appropriate for use with children aged 4–18 years, with higher scores indicating more autistic symptoms. The SRS forms can also be used when complete information regarding ASD diagnosis (ADOS; ADI-R) is missing. There are five sub-scales: social awareness (ability to recognize social cues), social cognition, social/reciprocal communication, social motivation interests, and stereotypy. The sum of all items is calculated to provide a total score (max of 195). T-scores are interpreted as follows: ≤59 T, within normal limits; 60–65 T, mild; 66–75 T, moderate; and ≥76 T, severe range. Cronbach’s alpha was 0.97. Global scores are calculated by averaging responses across items, with higher scores indicating more severe symptoms (max of 6) [39]. SRS-2 has been shown to be sensitive to changes in social functioning among children with ASD [40]. The scale was translated to Hebrew and is officially recommended for use with autistic children by the Ministry of Health [41]. The SRS-II was used at the initial assessment of the participants in the current research, enabling future research protocols a possibility to compare their participants’ level of autism with the participants of the current study.

### 4.4. Eligibility for Aquatic Intervention

This study included a hydrotherapy intervention, which requires a certain level of comfort in an aquatic environment. Participants and their parents were asked about their comfort in the water. In the eventuality that participants were not fully acclimated to an aquatic environment, the Humphries Assessment of Aquatic Readiness (HAAR) was used to assess eligibility for the hydrotherapeutic intervention. The HAAR, based on the principles of the Halliwick method, as well as components of popular aquatic assessments, is an evaluation used to measure one’s capabilities and functional performance in the water [42]. It takes into consideration the hydrotherapeutic needs and ability levels of people with various disabilities and is appropriate for use in ASD [9].

## 5. Statistical Analysis

All analyses were conducted with a significance threshold of α = 0.05. Normality of the dependent variables was first assessed using the Shapiro–Wilk test. Because the outcome variables (M-ABC-II domain scores and Total Test score) did not follow a normal distribution and instead exhibited a distributional pattern Poisson-like distribution (skewed, variance linked to mean), a repeated measures ANOVA was considered but rejected.

The primary inferential method was therefore Poisson regression with repeated measures, chosen to accommodate the non-normal distributional characteristics of the data and the repeated design across three time points (baseline, pre-intervention, post-intervention). Results are reported as Incidence Rate Ratios (IRR = exp(β)) with 95% confidence intervals, which quantify the relative change in performance following intervention.

Inter-rater reliability of the M-ABC-II scoring was evaluated using a Type C Intra-Class Correlation (ICC), based on a two-way mixed-effects model for consistency (ICC (3,1)). ICC values were computed from ANOVA mean squares (MSR, MSC, MSE) and are reported with point estimates and confidence intervals. This analysis served as a methodological quality check rather than an inferential test of treatment efficacy.

Finally, goal attainment scaling (GAS) scores were calculated according to the accepted statistical formula (Formula (1)) [37]. A mean GAS score of approximately 50 with a standard deviation of 10 is considered an indicator of appropriate goal setting and successful attainment and was used here as a complementary measure of functional improvement.

## 6. Results

### 6.1. M-ABC-II

The calculation regarding the M-ABC-II results refers to Standard Scores as these provide a measure of how far a participant’s raw score is from the average (mean) score of a normative population, we also used present results by percentiles, as these indicate the percentage of a participant in a normative sample and provide an easy-to-understand measure of a child’s relative standing compared to their peers.

Inter-rater reliability was tested by using ICC when comparing the results of the percentile ranks of the three domains separately, as well as the total percentile score, between the scoring of the PI (MW) and the independent observer. The average ICC (3,1) was 0.968 with a 95% confidence from 0.932 to 1.0 (*p* < 0.001), suggesting very high inter-rater reliability for all tests. This value is consistent with previous studies that were conducted using the M-ABC-II [26,27].

Table 2 presents Poisson regression coefficients for the three evaluated domains (Manual Dexterity, Aiming and Catching, and Balance), each assessed using two scoring formats (Standard Score and Percentile).

In all models, the Baseline category serves as the Reference group and is therefore not displayed in the table: its regression coefficient is always 0 and its odds ratio (OR = Exp(β)) is always 1. Accordingly, only the values for the intercept, study group, and control group are shown.

This means that each β and OR reported in the table represents a direct comparison against the baseline. Positive β values and ORs greater than 1 indicate a higher likelihood of success relative to the baseline, while negative β values and ORs below 1 indicate a lower likelihood of success relative to the baseline.

Consistently, the study group demonstrates statistically significant advantages across all domains, whereas the control group shows significantly reduced odds of success. These findings highlight the overall effectiveness of the intervention in improving motor performance outcomes

**Figure 2:**
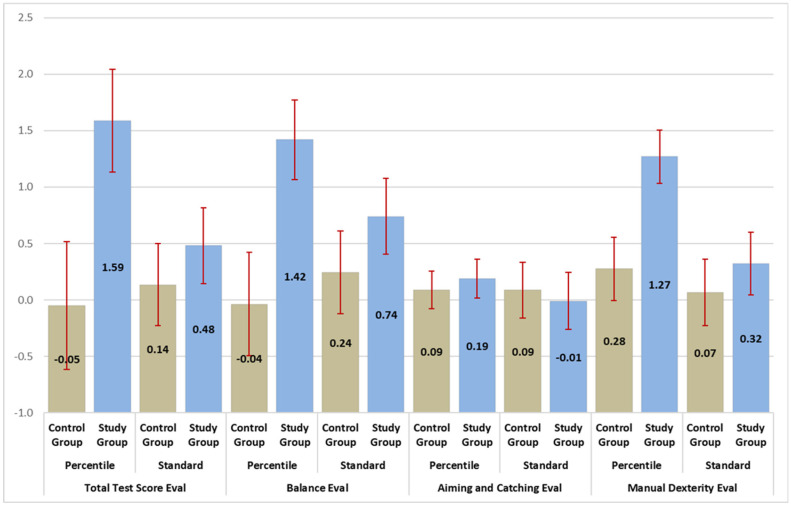
M-ABC-II, all fassonian coefficients in comparison to the baseline (no treatment).

Figure 2 presents a visual representation of the table above.

Table 3 divides the participants between the three age groups according to the M-ABC-II and indicates which subjects improved by moving up a category according to the M-ABC-II between their baseline average percentile score (pre-intervention) and percentile score of the third evaluation (post-intervention). These results were not statistically significant and inconclusive, due to the small subgroups.

**Figure 3:**
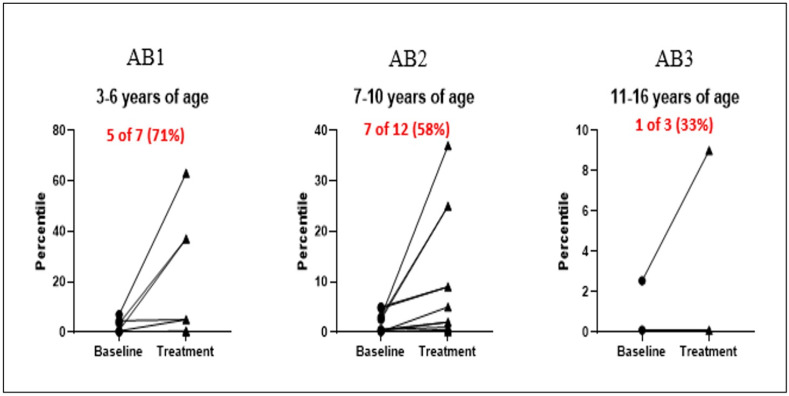
Number (%) of participants jumping a percentile, by age group.

Figure 3 shows that a 5/7 children (71%) of the younger age group moved up a category according to the M-ABC-II, while 7/12 (58%) of the middle age group and only 1/3 (33%) of the older age group children showed the same phenomenon (for better visual effect, age groups are written in black, improvement rates are in red color).

### 6.2. GAS 

**Figure 4:**
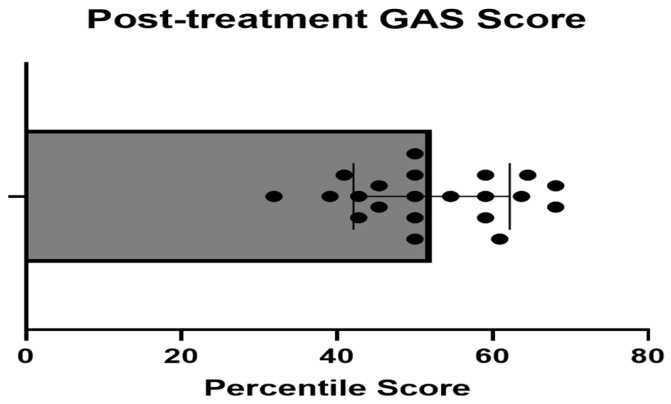
Post-intervention GAS scores.

The GAS score had a normal distribution, with a mean of 52.1 for this study, which implies improved function with the achievement of pre-established goals (see Figure 4 each dot in the Figure represents one participating child). Since 1 participant was not interested in participating in this part of the study, the average total GAS was based on 21 participants. Each point in Figure 4 represents the post-treatment score for each participant.

## 7. Discussion

This study focused on improving functional and motor abilities in children and adolescents with ASD and evaluated the impact of 12 weekly hydrotherapy sessions on the functional, balance, and coordination abilities of children diagnosed with ASD.

The results were based on a group of participants that were measured as their own comparison group to avoid the clinical diversity presented by all children with ASD. After a 12-session hydrotherapy intervention, the Total Score of the M-ABC-II improved by 5.8%, while the Balance and Coordination domain of the M-ABC-II increased significantly by 10.6% from the baseline measurements. Therefore, the main results suggest participants’ improvement during the intervention period, but not during the pre-intervention period. Upon examining secondary goals and sub-scales of the M-ABC-II, a significant improvement from baseline was also seen in the Manual Dexterity domain, while the Aiming and Catching domain (which was not a goal of the current intervention) showed no significant change. The mean GAS score for this study was 52.1, suggesting achievement of planned individual goals.

### 7.1. M-ABC-II Scores

The percentile and standard values of the Total Test score demonstrated statistically significant improvement from baseline, suggesting that a 12-session hydrotherapy intervention program, when performed by a licensed hydro-therapist, can yield functional improvements for children and adolescents with ASD.

The primary component of interest for this study, the Balance domain of the M-ABC-II, showed significant improvement from baseline. The importance of such a change is due to the fact that proper balance and postural control allow a person to participate in sports, kick a ball, and negotiate stairs [43]. In contrast, poor balance as a result of poor postural control, leads to a greater risk of falling, which affects the development of advanced motor skills and reduced participation. Moreover, adequate postural control enables a child to better manage educational activities and activities of daily living (ADL) [44]. Therefore, the authors adhere to the assumption that enhancing postural control (indicated by improved balance) might yield improved results for other functions [45]. A previous single case report [46] showed that balance and agility improved after a 10-week intervention of swimming training and water exercise, which is in line with our results. Recent meta-analysis and RCT evaluating motor improvements (among other areas) due to hydrotherapy intervention in children with ASD. Most of the improvements presented refer to in-water improvements (such as factors related to rotation, balance and control, and independent movement in water) [22,23], while the current intervention presented carryover from the hydrotherapy sessions to land improvements in balance and manual dexterity, which is a novel discovery, supporting previous reports [22,23] and extending the call of previous data related to the potential of hydrotherapy in improving motor skills of the ASD population.

Battaglia, in a study involving three participants diagnosed with low-functioning ASD, demonstrated that a 12-week aquatic program yielded improvement in running, hopping, and horizontal jumps [4]. As our study included 22 high-functioning participants, the combined results (which require further empirical support) suggest that this type of intervention might be promising for individuals with ASD at all functional levels.

Among the secondary domains that we scored, the Manual Dexterity domain also showed significant improvement from baseline. Manual dexterity, as defined by the Encyclopedia of Clinical Neuropsychology [47], is the ability to make coordinated hand and finger movements to grasp and manipulate objects. Functionally, it allows a person to perform precise movements (executive functions) such as writing, buttoning, and picking up small items. This skill develops primarily during childhood and can be affected by neurological disorders. Grip strength, which is considered an objective measure of upper extremity function, is essential for independence in early childhood functional tasks [48]. The findings suggest that improved manual dexterity can result from a motor-type intervention, such as hydrotherapy extending beyond the scope of the water to on-land improvements. Manual dexterity is also a function of core muscle stability, with coincides with the improvement in balance also achieved by the participants in the current study. These findings are in accordance with previous research [49].

No change was seen in the Aiming and Catching domain of the M-ABC-II. The current intervention was not aimed toward this goal, and we did not anticipate any change in this domain. This specific result strengthens our current findings, suggesting that using hydrotherapy to focus on specific treatment goals can yield positive results within the planned intervention goals. Previous research has suggested that water exercise programs can improve both social and aquatic skills in children with ASD [3,4,5,7,8,9]. A few of these studies addressed gross motor skills, but none explicitly measured manual dexterity or catching/throwing skills. One exception was a study by Battaglia that did not use hydrotherapy but included an aquatic intervention and demonstrated effectiveness for enhancing object control and locomotor skills, including tasks such as catching a ball with a tennis racket, stationary bounce, catching, kicking, and overhand throw [4].

### 7.2. GAS Scores

Scientific, valid, and reliable evaluation tools such as the M-ABC-II assess functions in specific areas and, therefore, reflect a child’s ability and achievements on a specific set of skills. However, they do not necessarily acknowledge the individuality of children with ASD, nor do they reflect the functional ability of the child within daily activities, thereby limiting assessment to the domains covered by the scale chosen by the researcher. Due to the individual clinical variability of children with ASD, a more individualized evaluation and approach that focuses on specific, individually tailored functional skills is warranted. The point of view of prioritizing personal variability among research participants has been promoted by the World Health Organization (WHO) with the presentation of the International Classification of Functioning (ICF) [50]. To address this issue, we used the GAS as a standardized test to establish individual functional goals for each participant, which were selected as important to each participant and/or their family [37]. Since the focus of this study was balance and coordination, the individual and functional goals were related. The functional goals established in cooperation with the children and their families revolved around playing sports, which included running, jumping, kicking, and/or hopping with the goal of improving the ability to play football, as well as running faster without falling. The average final GAS score was 52.1, and all but one child achieved their goals. The GAS has been used in prior studies related to hydrotherapy and other land-based exercise programs, specifically with children with Intellectual and Developmental Disability [51,52] and was suggested by others to be used as a measurement tool when assessing research outcomes with participants with ASD [53] in numerous situations; therefore, the current findings support the use of GAS within the ASD population and support its use in future intervention projects and within active therapeutic interventions in hydrotherapy and physical therapy [54,55].

### 7.3. Association Between Improvement and Patient Age

M-ABC-II scores can be quantified by the number of participants who moved up in percentile ranking categories, as well as the ages at which they did so. In the category of Balance, 11 children (50%) had improvements in the percentile ranking category. Five of seven children (71%) in the youngest age group AB1 (aged 3–6 y), five of twelve children (42%) in at age group AB2 (aged 7–10 y), while only one of three (33%) children in category AB3 (aged 11–16 y) moved up a category. These findings indicate that the youngest participants (<6) gained more from the intervention program than older participants. Despite these findings, our results were statistically inconclusive, probably as a result of a small group of participants within the AB3 group. Despite these inconclusive findings, previous evidence suggests that acquiring gross motor skills at the appropriate young age creates a solid foundation for further growth and can result in the development of more advanced milestones [56]. The current findings, in accordance with many previously reported intervention programs with children with ASD, suggest that early intervention is crucial for children with ASD, as it improves their overall prognosis [12,31,57]. Review articles published after the termination of the current study are also in alliance with the findings presented by the current study [58,59]

### 7.4. Additional Improvements as a Result of Hydrotherapy Intervention

Of note, while the hydrotherapy intervention was designed to improve Balance and Coordination, there was a significant improvement in the Manual Dexterity domain, but not in Aiming and Catching. A potential explanation for this could be that improved postural control reduces the need for participants to manage postural stability, thereby enabling them to direct their focus toward manual dexterity tasks [39,40,60], with a stable body acting as a proper base for limb-accurate activity. Alternatively, it is possible that with a longer hydrotherapy intervention, the Aiming and Catching domain would also have improved.

### 7.5. Qualitative Observation

The use of hydrotherapy as an intervention tool may result in improvements in other areas of function. Anecdotally, in recorded individual summation conversations, parents reported seeing improvement in other areas not directly connected with the direct goals of the current research. These improvements included general behavior, success at school, improved ability of participants to communicatively express themselves, improved ability to fall asleep at night, and an increased sense of independence, which was demonstrated by using public transportation independently for the first time. Numerous families reported improved self-confidence in social situations, including but not limited to initiating conversation and by the participants expressing their desires more coherently. Progress was also noted in school, specifically in terms of increased participation in class and better communication with the teacher. The oldest participant in the study acknowledged his difficulties with changes to his daily schedule and commented that now he feels he can “contain the fire”, while his mother said he is more flexible in his thinking and his ability to adapt to change. In addition, while he had difficulty getting out of bed in the morning, he is now able to get up immediately after he wakes up.

These anecdotal findings are supported by results from Mills et al. [61] as well as by recent meta-analysis and RCT’s [22,23], who demonstrated that children with ASD may benefit from a hydrotherapy program to improve their anxiousness and challenging behaviors, as well as reduce ill thoughts and overcome attentional difficulties. Other studies also acknowledged improvements in core ASD issues, such as social interactions and behaviors [7,9] as well as significantly decreased autistic behaviour in children with ASD [23] due to motor-type interventions including hydrotherapy [23,24].

### 7.6. Clinical Implications

The use of the M-ABC-II as a measuring tool was useful and is feasible as a measurement scale appropriate for measuring progress during a hydrotherapy intervention. This assumption coincides with previous suggestions by researchers exploring the field of ASD and motor-type interventions [31]. Due to the clinical versatility of individuals with ASD, we advise the use of the GAS when performing future hydrotherapy interventions for children and adolescents with ASD. The use of GAS allows assessment of the attainment of specific and personalized functional goals. The results of this study suggest that younger children (<6 years) experience greater improvements than older children. Despite their non-conclusiveness, these findings support the advancement of early motor-based intervention for children with ASD, in accordance with previous studies [12,31,56,57]. While the intervention itself was tailored to each individual participant, the hydrotherapeutic pool was occupied by other therapeutic sessions occurring concurrently. In some situations, there were up to 16 people (therapists and clients) working in parallel to the sessions of the study. This type of setting was found to disturb some of the participants and prevented them from fully cooperating, thereby preventing them from achieving their full potential. This suggests that children with ASD would gain more from an intervention in a more secluded therapeutic setting, as has been previously suggested [56]. The current results suggest that high-functioning children with ASD are inclined to achieve good results when introduced to a hydrotherapy program. Our clinical experience during the course of the current project suggests the need to construct favorable conditions for clients with ASD when involved in a therapeutic setting. For example, using a more secluded therapeutic environment (fewer surrounding interferences), an intensive intervention program (more therapeutic sessions per week), and a longer intervention period (more than 12 sessions). An important element that allowed this study to be successful was the relationship with the PI and the rapport built with the child and his family. Cooperation and trust between therapist and client are the cornerstones that encourage progress, especially with this population of children with ASD [62].

### 7.7. Limitations

Due to the clinical heterogeneity of children with autism, the authors found it inappropriate to select a different group of children (presenting a completely different picture of ASD despite having the same diagnosis) to serve as a control. Therefore, the same children were used as a control group (pre-intervention) and as a study group (with intervention).

While this study included a relatively larger number of participants than previous studies of hydrotherapy in ASD, a larger sample would allow us to draw better conclusions and more easily generalize them to the ASD population.

A more homogenous age group might yield more consistent results from which we can draw better conclusions, yet with less ability to generalize to the whole ASD spectrum.

While all participants were at the same diagnosed level of ASD, their individual conduct and ability to focus were important factors that affected their experience of the intervention. Cooperation could enhance or impede any treatment program, including hydrotherapy sessions.

Due to the specific nature of the hydrotherapy center (which serves ultra-orthodox clients, as well as the general population, the hours in the center are divided between the two genders, both therapists and clients). As the therapist was male, all the clients recruited for the current research had to be males as well. Therefore, the results of the current research’s findings can only be generalized to children and adolescents males with the diagnosis of ASD.

The exclusion of non-cooperating children narrowed the research population to include only high-functioning participants. Future research should also examine the results of a similar intervention program regarding less cooperating, low functioning, children with ASD.

## 8. Conclusions

The results of the current study demonstrated a positive impact of a 12-week hydrotherapy program; therefore, this type of intervention is suitable and recommended for this group of clients (high-functioning male children and adolescents with ASD). We recommend further studies on this topic should compare the interventions of hydrotherapy to other physical-motor type interventions, while also implementing hydrotherapy interventions with longer intervention period, with a larger sample population, including low-functioning children with ASD as well as females with the same diagnosis. As the incidence of ASD is constantly increasing worldwide, the necessity for continued research and intervention involving this group of clients is ever more important.

## Figures and Tables

**Table 1:** General data—all participants.

Participant #	Age in Months	SRS Score	GAS Goals	Initial M-ABC-II Total Score	Post-Intervention M-ABC-II Total Score
1	187	122		1	1
2	99	104	45.4	1	5
3	140	77	59.1	0.5	0.5
4	88	67	50	0.5	1
5	98		63.7	5	37
6	76	53	50	1	2
7	128	113	59.1	0.1	1
8	78	104	31.9	0.1	0.1
9	73	70	64.5	0.5	16
10	118	59	39.1	0.1	0.1
11	72	47	50	0.1	1
12	117	96	68.1	5	9
13	75	70	50	0.5	9
14	73	133	60.9	2	37
15	92	70	42.8	0.1	0.1
16	107	71	50	1	1
17	84	114	54.6	0.1	0.1
18	69	88	45.4	1	16
19	124	113	59.1	2	9
20	150	72	42.8	0.5	0.1
21	86	89	68.1	0.5	0.5
22	89	96	40.9	2	9
Mean	101.05	85.76	52.17	1.12	7.07
Min–Max (±SD)	189–69 (±59)	133–47 (±43)	68.1–31.9 (±18.1)	5–0.1 (±2.5)	37–0.1 (±18.5)
**Index**	SRS—Social Responsiveness Scale II		GAS—Goal attainment scaling		
	M-ABC-II—Movement Assessment Battery for Children—Second Edition				

**Table 2:** Comparison of all MABC-II domains within the study and control groups.

Dependent Variable	Score Type	Parameter	*β*	95% Wald Confidence Interval	Hypothesis Test	Exp(B) = OR	95% Wald Confidence Interval for Exp(B)		
				Lower	Upper	Wald *X^2^* Sig.		Lower	Upper
**Manual Dexterity Evaluation**	Standard	Intercept	1.363	1.152	1.575	0.000	3.909	3.164	4.829
		Study Group	0.325	0.047	0.602	0.022	1.384	1.049	1.826
		Control Group	0.067	−0.227	0.361	0.653	1.070	0.797	1.435
	Percentile	Intercept	1.533	1.324	1.742	0.000	4.632	3.758	5.708
		Study Group	1.269	1.032	1.505	0.000	3.557	2.808	4.506
		Control Group	0.277	−0.003	0.558	0.053	1.319	0.997	1.746
**Aiming and Catching Evaluation**	Standard	Intercept	1.705	1.527	1.883	0.000	5.500	4.602	6.573
		Study Group	−0.008	−0.261	0.244	0.949	0.992	0.770	1.277
		Control Group	0.087	-0.160	0.334	0.489	1.091	0.852	1.396
	Percentile	Intercept	2.561	2.439	2.683	0.000	12.950	11.465	14.627
		Study Group	0.189	0.018	0.360	0.030	1.208	1.018	1.434
		Control Group	0.091	−0.075	0.258	0.282	1.096	0.928	1.294
**Balance Evaluation**	Standard	Intercept	0.841	0.566	1.115	0.000	2.318	1.762	3.050
		Study Group	0.741	0.408	1.075	0.000	2.098	1.503	2.929
		Control Group	0.243	−0.124	0.609	0.195	1.275	0.883	1.839
	Percentile	Intercept	1.476	1.145	1.807	0.000	4.375	3.141	6.093
		Study Group	1.418	1.066	1.770	0.000	4.130	2.904	5.872
		Control Group	−0.036	−0.495	0.424	0.879	0.965	0.610	1.528
**Total Test Score Evaluation**	Standard	Intercept	0.916	0.652	1.181	0.000	2.500	1.919	3.256
		Study Group	0.481	0.145	0.817	0.005	1.618	1.156	2.265
		Control Group	0.136	−0.226	0.497	0.462	1.145	0.798	1.645
	Percentile	Intercept	0.742	0.314	1.170	0.001	2.100	1.369	3.221
		Study Group	1.587	1.131	2.043	0.000	4.889	3.099	7.713
		Control Group	−0.049	−0.615	0.517	0.866	0.952	0.541	1.677

**Table 3:** Age division of all participants.

AB	Participant #	Balance Baseline Average	Balance 3ed Evaluation Percentile	Change in Percentile Ranking
AB1 (3–6 yrs.)	11	0.1	0.5	No
	6	0.5	5	Yes
	18	0.75	37	Yes
	9	3.5	37	Yes
	13	4.55	5	Yes
	14	7	63	Yes
	8	0.1	0.1	No
AB2 (7–10 yrs.)	10	0.1	0.1	No
	12	0.1	5	Yes
	15	0.1	0.1	No
	17	0.1	0.5	No
	16	0.3	2	No
	4	0.5	2	No
	21	0.55	1	No
	7	2.5	25	Yes
	5	3	37	Yes
	22	3	25	Yes
	19	4.75	9	Yes
	2	5	9	No
AB3 (11–16 yrs.)	3	0.1	0.1	No
	20	0.1	0.1	No
	1	2.55	9	Yes

## Data Availability

Data is unavailable due to privacy restrictions.

## Supplementary Materials

The following supporting information can be downloaded at: https://www.mdpi.com/article/10.3390/children13010094/s1, Therapeutic protocol of the hydrotherapy program.

## References

[1] American Psychiatric Association What is Autism Spectrum Disorder? Updated August 2018. https://www.psychiatry.org/patients-families/autism/what-is-autism-spectrum-disorder.

[2] Centers for Disease Control and Prevention What is Autism Spectrum Disorder?. Updated March 2020. https://www.cdc.gov/autism/index.html.

[3] APTA Academy of Aquatic Physical Therapy. The Benefits of Aquatic Physical Therapy for Children. Updated 2021. https://aquaticpt.org/Files/Aquatic-Peds-Fact-Sheet.pdf.

[4] Battaglia G., Agro G., Cataldo P., Palma A., Alesi M. (2019). Influence of a specific aquatic program on social and gross motor skills in adolescents with autism spectrum disorders: Three case reports. J. Funct. Morphol. Kinesiol..

[5] Chu C.H., Pan C.Y. (2012). The effect of peer- and sibling-assisted aquatic program on interaction behaviors and aquatic skills of children with autism spectrum disorders and their peers/siblings. Res. Autism Spectr. Disord..

[6] Ennis E. (2011). The effects of a physical therapy-directed aquatic program on children with autism spectrum disorders. J. Aqua. Phy. Ther..

[7] Mortimer R., Privopoulos M., Kumar S. (2014). The effectiveness of hydrotherapy in the treatment of social and behavioral aspects of children with autism spectrum disorders: A systematic review. J. Multidiscip. Health.

[8] Pan C.Y. (2010). Effects of water exercise swimming program on aquatic skills and social behaviors in children with autism spectrum disorders. Autism.

[9] Pan C.Y. (2011). The efficacy of an aquatic program on physical fitness and aquatic skills in children with and without autism spectrum disorders. Res. Autism Spectr. Disord..

[10] Bhat A.N. (2020). Is motor impairment in autism spectrum disorder distinct from developmental coordination disorder? A report from the SPARK Study. Phys. Ther..

[11] Caçola P., Miller H.L., Williamson P.O. (2017). Behavioral comparisons in autism spectrum disorder and developmental coordination disorder: A systematic literature review. Res. Autism Spectr. Disord..

[12] Bhat A.N., Landa R.J., Galloway J.C. (2011). Current perspectives on motor functioning in infants, children, and adults with autism spectrum disorders. Phys. Ther..

[13] MacDonald M., Lord C., Ulrich D.A. (2013). The relationship of motor skills and social communicative skills in school-aged children with autism spectrum disorder. Adapt. Phys. Act. Q..

[14] Stins J.F., Emck C. (2018). Balance performance in autism: A brief overview. Front. Psychol..

[15] Castro-Sánchez A.M., Matarán-Peñarrocha G.A., Lara-Palomo I., Saavedra-Hernández M., Arroyo-Morales M., Moreno-Lorenzo C. (2012). Hydrotherapy for the treatment of pain in people with multiple sclerosis: A randomized controlled trial. Evid.-Based Complement. Altern. Med..

[16] Calandre E.P., Rodriguez-Claro M.L., Rico-Villademoros F., Vilchez J.S., Hidalgo J., Delgado-Rodriguez A. (2009). Effects of pool-based exercise in fibromyalgia symptomatology and sleep quality: A prospective randomized comparison between stretching and Ai Chi. Clin. Exp. Rheumatol..

[17] King M.R. (2016). Principles and application of hydrotherapy for equine athletes. Vet. Clin. Equine Pract..

[18] Torres-Ronda L., Del Alcázar X.S. (2014). The properties of water and their applications for training. J. Hum. Kinet..

[19] Geytenbeek J. (2002). Evidence for effective hydrotherapy. Physiotherapy.

[20] Pinto C., Salazar A.P., Marchese R.R., Stein C., Pagnussat A.S. (2019). The effects of hydrotherapy on balance, functional mobility, motor status, and quality of life in patients with parkinson disease: A systematic review and meta-analysis. J. Inj. Funct. Rehabil..

[21] Ansari S., Hosseinkhanzadeh A.A., AdibSaber F., Shojaei M., Daneshfar A. (2021). The effects of aquatic versus kata techniques training on static and dynamic balance in children with autism spectrum disorder. J. Autism Dev. Disord..

[22] Shariat A., Najafabadi M.G., Dos Santos I.K., Anastasio A.T., Milajerdi H.R., Hassanzadeh G., Nouri E. (2024). The Effectiveness of Aquatic Therapy on Motor and Social Skill as Well as Executive Function in Children with Neurodevelopmental Disorder: A Systematic Review and Meta-analysis. Arch. Phys. Med. Rehabil..

[23] Van t Hooft P., Moeijes J., Hartman C., Van Busschbach J., Hartman E. (2024). Aquatic Interventions to Improve Motor and Social Functioning in Children with ASD: A Systematic Review. Rev. J. Autism Dev. Disord..

[24] Griffiths A., Toovey R. (2018). Psychometric properties of gross motor assessment tools for children: A systematic review. BMJ Open.

[25] Palisano R., Orlin M., Schreiber J. (2016). Campbell’s Physical Therapy for Children Expert Consult.

[26] Downs S.J., Boddy L.M. (2020). Motor competence assessments for children with intellectual disabilities and/or autism: A systematic review. BMJ Open Sport Exerc. Med..

[27] Cancer A., Minoliti R. (2020). Identifying Developmental Motor Difficulties: A Review of Tests to Assess Motor Coordination in Children. J. Funct. Morphol. Kinesiol..

[28] Rebelo M., Serrano J. (2021). Evaluation of the Psychometric Properties of the Portuguese Peabody Developmental Motor Scales-2 Edition: A Study with Children Aged 12 to 48 Months. Children.

[29] Baharudin N.S., Harun D. (2020). An Assessment of the Movement and Function of Children with Specific Learning Disabilities: A Review of Five Standardised Assessment Tools. Malays. J. Med. Sci..

[30] Ellinoudis T., Evaggelinou C., Kourtessis T., Konstantinidou Z., Venetsanou F., Kambas A. (2011). Reliability and validity of age band 1 of the movement assessment battery for children–second edition. Res. Dev. Disabil..

[31] Liu T., Breslin C.M. (2013). Fine and gross motor performance of the MABC-2 by children with autism spectrum disorder and typically developing children. Res. Autism Spectr. Disord..

[32] Schoemaker M.M., Niemeijer A.S., Flapper B.C., Smits-Engelsman B.C.M. (2012). Validity and reliability of the movement assessment battery for children-2 checklist for children with and without motor impairments. Dev. Med. Child Neurol..

[33] Engel-Yeger B., Rosenblum S., Josman N. (2010). Movement assessment battery for children (M-ABC): Establishing construct validity for Israeli children. Res. Dev. Disabil..

[34] Griffiths A., Morgan P., Anderson P.J., Doyle L.W., Lee K.J., Spittle A.J. (2017). Predictive value of the movement assessment battery for children-second edition at 4 years, for motor impairment at 8 years in children born preterm. Dev. Med. Child Neurol..

[35] Jaikaew R., Satiansukpong N. (2019). Movement assessment battery for children-second edition (MABC2): Cross-cultural validity, content validity, and interrater reliability in thai children. Occup. Ther. Int..

[36] Holm I., Tveter A.T., Aulie V.S., Stuge B. (2013). High intra- and inter-rater chance variation of the movement assessment battery for children 2, age band 2. Res. Dev. Disabil..

[37] Turner-Stokes L. (2009). Goal attainment scaling (GAS) in rehabilitation: A practical guide. Clin. Rehabil..

[38] Constantino J.N., Gruber C.P. (2012). Social Responsiveness Scale–Second Edition (SRS-2).

[39] Lang K., Larsson E., Mavromara L., Simic M., Treasure J., Tchanturia K. (2016). Diminished facial emotion expression and associated clinical characteristics in anorexia nervosa. Psych. Res..

[40] Wood J.J., Drahota A., Sze K., Van Dyke M., Decker K., Fujii C., Bahng C., Renno P., Hwang W.-C., Spiker M. (2009). Brief Report: Effects of Cognitive Behavioral Therapy on Parent-Reported Autism Symptoms in School-Age Children with High-Functioning Autism. J. Autism Dev. Disord..

[41] Department of Child Development and Rehabilitation, Ministry of Health (2023). An On-Line Guide For Child Development Units. https://www.gov.il/en/departments/units/child_development_and_rehabilitation_unit/govil-landing-page.

[42] Humphries K.M. (2008). Humphries’ Assessment of Aquatic Readiness. Master’s Thesis.

[43] Fong S.S.M., Chung L.M.Y., Bae Y.H., Vackova D., Ma A.W.W., Liu K.P.Y. (2018). Neuromuscular processes in the control of posture in children with developmental coordination disorder: Current evidence and future research directions. Curr. Dev. Disord. Rep..

[44] Fong S.S.M., Tsang W.W.N., Ng G.Y.F. (2012). Altered postural control strategies and sensory organization in children with developmental coordination disorder. Hum. Mov. Sci..

[45] Westcott S.L., Burtner P. (2010). Postural control in children: Implications for pediatric practice. Phys. Occup. Ther. Pediatr..

[46] Yilmaz I., Yanardag M., Birkan B., Bumin G. (2004). Effects of swimming training on physical fitness and water orientation in autism. Pediatr. Int..

[47] Makofskee B., Kreutzer J.S., DeLuca J., Caplan B. (2018). Manual dexterity. Encyclopedia of Clinical Neuropsychology.

[48] Abu-Dahab S.M.N., Skidmore E.R., Holm M.B., Rogers J.C., Minshew N.J. (2012). Motor and tactile-perceptual skill differences between individuals with high-functioning autism and typically developing individuals ages 5–21. J. Autism Dev. Disord..

[49] Hassani F., Shahrbanian S., Shahidi S.H., Sheikh M. (2022). Playing games can improve physical performance in children with autism. Int. J. Dev. Disabil..

[50] McDougall J., Wright V. (2009). The icf-cy and goal attainment scaling: Benefits of their combined use for pediatric practice. Disabil. Rehabil..

[51] Jones M.C., Walley R.M., Leech A., Paterson M., Common S., Metcalf C. (2006). Using goal attainment scaling to evaluate a needs-led exercise program for people with severe and profound intellectual disabilities. J. Intellect. Disabil..

[52] Materne M., Frank A., Arvidsson P. (2021). The utility of goal attainment scaling in evaluating a structured water dance intervention for adults with profound intellectual and multiple disabilities. Heliyon.

[53] Staunton H., McIver T., Tillmann J., Clinch S., Hanrahan V., Ewens B., Averius C., Barsdorf A.I., Baranger A., Berry Kravis E. (2025). Development of a Goal Attainment Scale (GAS) outcome measure for clinical interventional studies in paediatric autism. Autism.

[54] Lee C.E., Shogren K.A., Segal J., Pezzimenti F., Aleman-Tovar J., Taylor J.L. (2021). Goal attainment scaling—Community-based: A method to incorporate personalized outcomes into intervention research with youth and adults on the autism spectrum. Autism.

[55] Ruble L., McGrew J., Dale B., Yee M. (2022). Goal attainment scaling: An idiographic measure sensitive to parent and teacher report of IEP goal outcome assessment for students with ASD. J. Autism Dev. Disord..

[56] MacDonald M., McIntyre L.L. (2019). The relationship of age early motor skills and observable child behaviors in young children with developmental delays. Res. Dev. Disabil..

[57] National Institute of Child Health and Human Development Early Intervention for Autism. Updated 19 April 2021. https://www.nichd.nih.gov/health/topics/autism/conditioninfo/treatments/early-intervention.

[58] Vodakova E., Chatziioannou D., Jesina O., Kudlacek M. (2022). The Effect of Halliwick Method on Aquatic Skills of Children with Autism Spectrum Disorder. Int. J. Environ. Res. Public Health.

[59] Movahedi A., Bahrami F., Marandi S.M., Abedi A. (2013). Improvement in social dysfunction of children with autism spectrum disorder following long term kata techniques training. Res. Autism Spectr. Disord..

[60] Mills W., Kondakis N., Orr R., Warburton M., Milne N. (2020). Does Hydrotherapy Impact Behaviours Related to Mental Health and Well-Being for Children with Autism Spectrum Disorder? A Randomised Crossover-Controlled Pilot Trial. Int. J. Environ. Res. Public Health.

[61] Brewe A.M., Mazefsky C.A., White S.W. (2021). Therapeutic alliance formation for adolescents and young adults with autism: Relation to treatment outcomes and client characteristics. J. Autism Dev. Disord..

[62] Cools W., Martelaer K.D. (2009). Movement skill assessment of typically developing preschool children: A review of seven movement skill assessment tools. J. Sports Sci. Med..

