# Supplementary material for: Balance and Coordination Improvements in Children and Adolescents with Autism Spectrum Disorder (ASD), Resulting from a Hydrotherapy Intervention
Source: Children (Basel). 2026 Jan 8;13(1):94. doi: 10.3390/children13010094 (PMC12840020; doi:10.3390/children13010094)
Supplement: Supplementary file 1 [file children-13-00094-s001.zip › children-3873827-supplementary.pdf]

Therapeutic protocol of the hydrotherapy program

A series of exercises supporting balance and motor coordination were constructed. Each exercise can be performed at several levels of difficulty. These exercises were performed during every hydrotherapy session, by each participant, with the intent of reaching the highest difficulty level possible for each exercise, based on their functional abilities.

Balance

Exercise 1 -

Initial position: Begin with the child standing with the water depth at waist height, with his feet at a wide base of support (BOS) with the goal of reaching a closed BOS (feet together).

Changing difficulty levels:

1) Difficulty level I - Throughout the varying distances of BOS, the child is asked to reach his hands out to rings that are held out at varying heights and distances. The goal is for the child to take those rings while maintaining balance within the current BOS. The child will be asked to pick up rings floating on the water using the hand on the side of the ring.
2) Difficulty level II - The child stands with the same BOS with water at chest level.
3) Difficulty level III - therapist creates small waves with his hands or a Frisbee.
4) Difficulty level IV - The child reaches with the hand on opposite side, or reaches with both hands together.
5) Difficulty level VI - The therapist holds the rings at varying heights and distances to encourage movement in varying planes of movement (with the incorporation of rotation being the most challenging).
6) Difficulty level VII - Have the child perform the same exercises when standing on a flotation device.

Exercise 2 -

Initial position - Begin with the child standing with the water depth at waist height, while standing on one foot, with a floatation device attached to the other foot, while the therapist measures the duration of maintaining this position.

Changing difficulty levels:

1) Difficulty level I - The child’s hands could be held on the water, helping him to maintain the one-legged position.
2) Difficulty level II - Arms crossed in front of their body.
3) Difficulty level III - The child's hands on his hips.

4) Difficulty level IV - The child's hands are clasped and held over his head.
5) Difficulty level V - The child moves his arms between the various positions mentioned in the previous levels of difficulty, while maintaining the current standing position.

Exercise 3 -

Initial position - The child begins by sitting, and maintaining a sitting position, on a large square floatation mattress, while the movement of the water creates a natural perturbation. The therapist gently holds the mattress to prevent it from creating large perturbations.

Changing difficulty level

1) Difficulty level I - The child is asked to transition into, and maintain, a knee-standing position.
2) Difficulty level II - The child is asked to transition into and maintain a half-kneeling position.
3) Difficulty level III - The child is asked to transition into and maintain a standing position.
4) Difficulty level IV - The child is asked to transition into and maintain all previous positions with the therapist creating stronger perturbations by manually moving the floatation mattress in one direction, while the child maintains the position.
5) Difficulty level V - The child is asked to transition into and maintain all previous positions, with the therapist creating stronger perturbations by manually slowly moving the floatation mattress in varying planes of movement (straight, sideways, circular), while the child maintains the position.
6) Difficulty level VI - The child is asked to transition into and maintain all previous positions, with the therapist creating stronger perturbations by manually quickly moving the floatation mattress in varying planes of movement (straight, sideways, circular), while the child maintains the position. This could be done slowly or quickly, and with or without warning.
7) Difficulty level VII - All perturbations (which will be modulated based on the response and reaction of the child) are performed during the actual transitioning between positions.

Exercise 4 -

Initial position - The child is helped into a standing position, with both feet on a noodle (without toes or heels touching the floor), with the BOS slightly wider than the width of the pelvis, and with the water at waist height, while the natural movement of the water causes a natural perturbation. The child is timed to see how long the position can be maintained.

1) Difficulty level I - While the child maintains the current position, stronger perturbations are applied by the therapist with the use of a Frisbee against the water.
2) Difficulty level II - The child closes the BOS by standing with his feet together.
3) Difficulty level III - While the child stands with his feet together, stronger perturbations are applied by the therapist with the use of a Frisbee against the water.

Exercise 5 -

Initial position - The child is helped into a sitting position on a noodle in the frontal plane with both feet on the ground, while holding on to the sides/ends of the noodle with each hand.

1) Difficulty level I - The child is asked to sit without holding the noodle with his hands, while his feet are still in contact with the floor.
2) Difficulty level II - Perturbations are applied while the child sits without holding the noodle, while his feet are in contact with the floor.
3) Difficulty level III - The child sits on the noodle, while his feet are no longer in contact with the floor, holding the noodle with his hands.
4) Difficulty level IV - Perturbations are applied while the child sits on the noodle, with his feet no longer in contact with the floor, and holding the noodle with his hands.
5) Difficulty level V - The child maintains the sitting position, while his feet are not in contact with the floor, and without holding the noodle.
6) Difficulty level VI - Perturbations are applied as the child maintains the sitting position, while his feet are not in contact with the floor, and without holding the noodle.

Motor Coordination

Exercise 1 -

Initial position - Sitting on the steps of the pool with the water slightly below shoulder level, and proceeding to move arms in the pattern of breast stroke.

1) Difficulty level I - The child moves his legs in the pattern of breast stroke.
2) Difficulty level II - The child moves his arms and legs in a coordinated pattern of the breast stroke, while remaining in the seated position.
3) Difficulty level III - The child leans forward while sitting, and moves his arms in the pattern of breast stroke, while coordinating putting his head in and out of the water as part of the pattern of movement.
4) Difficulty level IV - While sitting, the child combines the arm and leg movements, while putting his head in and out of the water.

5) Difficulty level V - While walking, the child moves his arms in the pattern of breast stroke, while coordinating putting his head in and out of the water, as part of the pattern of movement.
6) Difficulty level VI - While floating on his stomach on a noodle placed under the upper chest, the child moves his legs in the pattern of breast stroke.
7) Difficulty level VII - While floating on his stomach on a noodle placed under the upper chest, the child combines the arm and leg movements, while putting his head in and out of the water.

Exercise 2 -

Initial position - The child walks in water at waist level along the width of the pool, lifting each knee out of the water, and touching the knee with the opposite hand, while maintaining proper posture (straight back, head upright while looking forward).

1) Difficulty level I - The child continues the same gait pattern, while balancing a toy, ring, or cup of water on his head.
2) Difficulty level II - The child continues with same gait pattern as level I of difficulty, while wearing flippers.
3) Difficulty level III - While holding a noodle at its ends, the child walks in water at waist level along the width of the pool. As he lifts each knee out of the water, he closes the ends of the noodle under his knee. This is repeated with each step. Proper posture (straight back, head upright while looking forward) must be maintained.
4) Difficulty level IV - The child continues the same gait pattern as level III of difficulty, while balancing a toy, ring, or cup of water on their head. Flippers can then be added.
5) Difficulty level V - The child walks in water at waist level along the width of the pool, lifting each knee out of the water and passing a ball under the axilla of his knee/lower thigh, from the opposite hand to the hand on side of the leg being lifted. The ball is always passed under the leg from the inside to the outside. Proper posture (straight back, head upright while looking forward) must be maintained.
6) Difficulty level VI - The child continues the same gait pattern as level V of difficulty, while balancing a toy, ring, or cup of water on their head. Flippers can then be added.
7) Difficulty level VII - The child begins by standing in water at waist level. His gait pattern begins by stepping down into half kneel, transitioning forward back into standing, followed by stepping into half kneel with the other leg, and continuing with this pattern.

8) Difficulty level VIII - The child continues the same gait pattern as level VII of difficulty, while balancing a toy, ring, or cup of water on his head.
9) Difficulty level IX - The child walks in water at waist level, while balancing items on a tray/board without having them fall. These items could range from simple toys that have a wide base and are easy to balance, to a ball, to a cup of water that is filled with varying volumes, and not letting the water spill.

Exercise 3

Initial position - The child stands in water at waist level with his BOS the width of his pelvis. Rings are thrown to the child from a distance of 2 meters, and he catches them by threading his hand through the ring as it approaches.

1) Difficulty level I - Child stands on a narrow BOS, with his feet together.
2) Difficulty level II - The rings are thrown slightly out of his BOS.
3) Difficulty level III - The same exercise is done from a distance of 4 meters.
4) Difficulty level IV - Child is on the floatation mattress in a fully kneeling position.
5) Difficulty level V - Child is standing on the floatation mattress.
6) Difficulty level VI - Child is on the floatation mattress in a half-kneeling position.

Exercise 4 -

As an extension to exercise 4 in the Balance section,

Initial position - The child is helped into a standing position, with both feet on a noodle (without toes or heels touching the floor), with the BOS slightly wider than the width of the pelvis, and with the water at waist height, while the natural movement of the water causes a natural perturbation. The child is timed to see how long the position can be maintained.

1) Difficulty level I - While the child maintains the current position, stronger perturbations are applied by the therapist with the use of a Frisbee against the water.
2) Difficulty level II - The child closes the BOS by standing with his feet together.
3) Difficulty level III - While the child stands with his feet together, stronger perturbations are applied by the therapist with the use of a Frisbee against the water.

The child performs the same task with the following changes in levels of difficulty:

1) Difficulty level I - The child is helped onto the noodle with one foot only, while he has to bring the other foot to the noodle without assistance.
2) Difficulty level II - The child receives no assistance getting on the noodle.
